# Semantic fluency in deaf children who use spoken and signed language in comparison with hearing peers

**DOI:** 10.1111/1460-6984.12333

**Published:** 2017-07-10

**Authors:** C. R. Marshall, A. Jones, A. Fastelli, J. Atkinson, N. Botting, G. Morgan

**Affiliations:** ^1^ UCL Institute of Education University College London London UK; ^2^ UCL Deafness Cognition and Language Research Centre University College London London UK; ^3^ University of Padua Padua Italy; ^4^ Language and Communication Science School of Health Sciences City University of London London UK

**Keywords:** deaf, semantic fluency, vocabulary, lexicon, executive functions, British Sign Language (BSL)

## Abstract

**Background:**

Deafness has an adverse impact on children's ability to acquire spoken languages. Signed languages offer a more accessible input for deaf children, but because the vast majority are born to hearing parents who do not sign, their early exposure to sign language is limited. Deaf children as a whole are therefore at high risk of language delays.

**Aims:**

We compared deaf and hearing children's performance on a semantic fluency task. Optimal performance on this task requires a systematic search of the mental lexicon, the retrieval of words within a subcategory and, when that subcategory is exhausted, switching to a new subcategory. We compared retrieval patterns between groups, and also compared the responses of deaf children who used British Sign Language (BSL) with those who used spoken English. We investigated how semantic fluency performance related to children's expressive vocabulary and executive function skills, and also retested semantic fluency in the majority of the children nearly 2 years later, in order to investigate how much progress they had made in that time.

**Methods & Procedures:**

Participants were deaf children aged 6–11 years (*N* = 106, comprising 69 users of spoken English, 29 users of BSL and eight users of Sign Supported English—SSE) compared with hearing children (*N* = 120) of the same age who used spoken English. Semantic fluency was tested for the category ‘animals’. We coded for errors, clusters (e.g., ‘pets’, ‘farm animals’) and switches. Participants also completed the Expressive One‐Word Picture Vocabulary Test and a battery of six non‐verbal executive function tasks. In addition, we collected follow‐up semantic fluency data for 70 deaf and 74 hearing children, nearly 2 years after they were first tested.

**Outcomes & Results:**

Deaf children, whether using spoken or signed language, produced fewer items in the semantic fluency task than hearing children, but they showed similar patterns of responses for items most commonly produced, clustering of items into subcategories and switching between subcategories. Both vocabulary and executive function scores predicted the number of correct items produced. Follow‐up data from deaf participants showed continuing delays relative to hearing children 2 years later.

**Conclusions & Implications:**

We conclude that semantic fluency can be used experimentally to investigate lexical organization in deaf children, and that it potentially has clinical utility across the heterogeneous deaf population. We present normative data to aid clinicians who wish to use this task with deaf children.

**What this paper adds**

**What is already known on the subject**

The semantic fluency task, particularly involving the semantic category ‘animals’, is widely used as a research and clinical tool across the lifespan. Little is known, however, about how deaf children perform on this task, or whether there are differences between deaf children who use spoken language and those who sign.

**What this paper adds to existing knowledge**

Our study of 106 deaf children aged 6–11 years from the UK revealed that deaf children on average produced fewer responses compared with hearing children, although there was substantial overlap between the two groups. There were also similarities in the two groups’ patterns of performance, suggesting that the task measures the same cognitive processes in both groups, regardless of the language that the deaf children responded in (BSL, spoken English or SSE).

**What are the potential or actual clinical implications of this work?**

The data from this study, which investigates semantic fluency in the largest sample of deaf children to date, suggest that semantic fluency could have value both as a research tool for investigating deaf children's vocabulary and executive functions, and as a clinical assessment tool. The normative data for deaf children aged 6–11 years that are included in this paper will aid clinicians to use the task with deaf children in that age range.

## Introduction

Deafness impacts adversely on children's ability to process and acquire spoken languages. Signed languages provide a more easily accessible language input, and for the small proportion of deaf children who are born to deaf signing parents (‘native signers’) signed language development can proceed with very similar milestones and timescale to spoken language acquisition in hearing children (Anderson and Reilly 2002, Mayberry and Squires 2006, Newport and Meier 1985). However, the vast majority of deaf children—approximately 95%—are born to hearing parents who do not sign (Mitchell and Karchmer 2004) and so they do not usually have access to sign language, at least during the early stages of language acquisition (Lu *et al*. 2016). Deaf children as a group are therefore at high risk of language delays. This in turn has implications for other areas of development, and lower academic achievement and poorer social, emotional and mental well‐being outcomes are reported (Convertino *et al*. 2009, Vaccari and Marschark 1997, van Eldik *et al*. 2004).

This paper focuses on vocabulary, a fundamental part of language whose development is closely related to the development of grammar, narrative ability and literacy (Duff *et al*. 2015, Fenson *et al*. 1994, Lee 2011, Paul *et al*. 1997). There is considerable variability in the rate of vocabulary development even in hearing children (Duff *et al*. 2015, Fenson *et al*. 1994), but this variability is particularly marked in the case of deaf children, and is increased by heterogeneity in communication approaches and quality of language input. Native signers generally outperform non‐native signers on measures of sign vocabulary (Hermans *et al*. 2008, Schick and Hoffmeister 2001), but even native signers have been shown to know fewer lexical items than hearing children (Rinaldi *et al*. 2014). Deaf children who use spoken language also tend to have lower vocabulary levels than their hearing peers (Convertino *et al*. 2014, Yoshinaga‐Itano *et al*. 2010, Ziv *et al*. 2013). Even though rapid advances in hearing technologies such as hearing aids and early cochlear implantation generally yield good progress in improving deaf children's access to the sounds of spoken language (Yoshinaga‐Itano *et al*. 2010), many deaf children still do not reach age‐equivalent vocabulary capabilities for either expressive or receptive vocabulary (see Lund 2016 for a recent meta‐analysis).

Children's vocabulary abilities can be investigated in different ways. In this study we used the semantic fluency task, which has been employed to investigate lexical organization and retrieval across the lifespan. Semantic fluency requires participants to name as many exemplars as they can from a particular semantic category (such as ‘foods’, ‘animals’ or ‘household objects’) in a limited period of time. Given the limited time for responding (most usually just 1 min), the task does not provide an exhaustive list of the words that a participant knows, but it does reveal those words that come most readily to mind.

The semantic fluency task provides a measure of two things: lexical organization and executive functions (EFs; Ardila *et al*. 2006, Bose *et al*. 2017). With respect to lexical organization, if participants can generate exemplars in response to a superordinate label, e.g., ‘animals’, then this suggests that their semantic knowledge is organized taxonomically. When a word is spoken (or signed), it is assumed that this will in turn activate other words or concepts that are semantically similar or related to it. Hence, it is also assumed that the order in which words are produced will indicate, indirectly, their proximity to each other in the lexicon. Characteristic findings for this task are that items are produced in clusters of semantically related words (e.g., ‘farm animals’, ‘pets’, ‘sea animals’), and that more prototypical category exemplars are produced more frequently than less typical ones (see Marshall *et al*. 2013 for a review of the relevant literature). With respect to EFs, the task requires the use of word‐retrieval strategies, which in turn rely on executive abilities, namely cognitive flexibility (i.e., set‐shifting between different clusters), working memory (to keep track of items that have already been produced), and inhibition (so as to avoid repeating previous responses, and responses that are not relevant to the category) (Rosen and Engle 1997). Overall, optimal performance on the semantic fluency task requires a systematic search of the mental lexicon, word retrieval within a subcategory (e.g., ‘farm animals’), and, when a subcategory is exhausted, switching to a new subcategory (e.g., ‘pets’) (Troyer *et al*. 1997).

Semantic fluency is widely used in studies of the lexicon in both children and adults, and as part of neuropsychological test batteries to assess language and cognitive impairment. Its simple instructions mean that it can be administered to a wide range of participant groups. Ardila *et al*. (2006) argue that the task, and in particular the category ‘animals’, meets criteria for clinical usefulness (i.e., specific patterns of performance and error types are associated with specific brain pathologies), experimental usefulness (it has been used experimentally in non‐clinical populations, and the pattern of brain activation correlated with performance is well known), and psychometric validity (performance on it correlates with performance on other assessments). Furthermore, Ardila *et al*. argue that ‘animals’ is a semantically clear category across speakers of different languages and living in different countries.

Given deaf children's delayed vocabulary and delayed EF development as measured by tasks of cognitive flexibility, working memory, inhibition and planning (Botting *et al*. 2016, Figueras *et al*. 2008), they are predicted to perform worse on the semantic fluency task compared with same‐age hearing children. To date, however, there have been very few studies to investigate whether this is indeed the case.

One exception is Wechsler‐Kashi *et al*. (2014), who used the spoken semantic fluency task with 20 deaf American children aged 7–10 years who had received cochlear implants (CIs) and who were learning spoken language, and 20 hearing children matched for age and non‐verbal IQ. The deaf children produced significantly fewer responses compared with typically developing children. For the deaf children, age at implantation and years of CI use were significantly correlated with the number of responses: children who had been implanted earlier retrieved more words, and children who had used their implants for a longer duration of time also tended to retrieve more words. There were no differences between deaf and hearing children with respect to the more qualitative aspects of performance, namely the number of clusters, number of switches, or mean cluster size. Nevertheless, an analysis with a slightly larger sample (*n* = 27 deaf and *n* = 27 hearing; Kenett *et al*. 2013) found that there were differences between the two groups in the semantic network for ‘animals’: fewer different animal names were provided by the deaf group as a whole compared with the hearing group, and the semantic network of the deaf children was more condensed and less spread out. The semantic network of the deaf group was therefore argued to be under‐developed compared with that of the hearing children (Kenett *et al*. 2013).

For children who use a signed language, there are only two published studies to our knowledge: Marshall *et al*. (2013) in British Sign Language (BSL) and Beal‐Alvarez and Figueroa (2017) in American Sign Language (ASL). Marshall *et al*. (2013) tested 35 deaf children aged 4–15 years, 13 of whom had been identified as having a specific language impairment (SLI) which manifested in their use of BSL. The categories used were ‘animals’ and ‘food’. The performance of these deaf signers was very similar to that reported for hearing children in spoken languages, with children producing similar clusters and switching between clusters, and producing the same prototypical responses that have been noted in the spoken language literature. Productivity increased with age. Interestingly, the results of the children with and without SLI were comparable in most respects, but the group with SLI made occasional word‐finding errors and gave fewer responses in the first 15 s. Marshall *et al*.’s results suggest that semantic fluency can be used with deaf children who sign, that it is a valid measure of their lexical organization and retrieval, and that it might be clinically sensitive in that population. An important limitation of that study, was, however, the lack of a hearing comparison group. Marshall *et al*. (2013: 215) noted that the number of responses was within the range reported for hearing children in spoken languages, but they did not test this directly with an age‐matched hearing group.

Beal‐Alvarez and Figueroa (2017) employed the animal semantic fluency task in ASL with deaf children in the United States and Puerto Rico. Like Marshall *et al*. (2013) for BSL, Beal‐Alvarez and Figueroa (2017) report clustering of responses around subcategories such as ‘pets’, ‘water animals’ and ‘farm animals’, and they too found an increase in productivity with age. Some of their participants had additional diagnoses of, for example, autism or mild or moderate intellectual disability, and such children performed more poorly than their typically developing deaf peers: they produced fewer correct items and made more errors (such as non‐animal signs) during the task. Again, this pattern of findings suggests that the semantic fluency task is sensitive to language and cognitive impairments in deaf signers. However, as was the case for Marshall *et al*.’s (2013) study, Beal‐Alvarez and Figueroa (2017) did not include a hearing comparison group.

Thus, recent studies of semantic fluency in deaf children have been valuable, but the sample sizes are small and there are several questions that remain relatively unexplored within the heterogeneous population of deaf children that includes those who sign and who use spoken language:

- How does the semantic fluency performance of deaf children compare with that of hearing children, and does it differ between groups of deaf children who sign or use spoken language to communicate?
- How does semantic fluency performance relate to children's expressive vocabulary and EFs?
- Do any group differences between deaf and hearing children's semantic fluency performance persist as they get older?

If the semantic fluency task is to be useful as a clinical and experimental tool in the deaf population these questions need to be investigated for both signed and spoken language.

## Methods

### Participants

Participants were 226 children (106 deaf, 120 hearing) living in the UK and Ireland and who had English, BSL or Sign‐Supported English (SSE; i.e., the simultaneous use of sign and spoken English) as their primary method of communication. None of the children had any known developmental disorders such as autism, attention deficit/hyperactivity disorder (ADHD) or cerebral palsy. They had previously been recruited as part of a larger sample in order to study the relationship between language and EFs in deaf and hearing children. Language and EF data from the majority of that group have been presented by Botting *et al*. (2016), who did not present the semantic fluency data that are the focus of the current paper. Data from seven deaf and 11 hearing participants of Botting *et al*.’s group were not used here because they did not do the semantic fluency task, while data from an additional group of five deaf and six hearing children were not included in Botting *et al*. but were tested as part of the same study and are included here. The groups in both studies therefore overlap to a very high degree. To gain a sample that is representative of deaf children's varied educational and language experiences, deaf participants were recruited from both specialist deaf (day and residential schools) and mainstream schools (with and without a specialist hearing unit).

Table 1 provides details of participants’ hearing status (deaf or hearing), gender, age and deaf group membership. Group membership was defined according to the language in which participants completed the semantic fluency task and the Expressive One‐Word Picture Vocabulary Test (Brownell 2000), and which was either BSL, spoken English or SSE; BSL users were then subgrouped according to whether they were native or non‐native signers. The deaf group as a whole was well‐matched to the hearing group for age, *t*(224) = 0.342, *p* = .746. On a test of non‐verbal cognitive ability (the matrix reasoning subtest of the Wechsler Abbreviated Scale of Intelligence; Wechsler 1999), the mean *T*‐score of the deaf group was 50.21 (SD = 10.47) and of the hearing group was 54.50 (9.74). The deaf group therefore scored within the normal range (mean = 50, SD = 10), but an independent samples *t*‐test nevertheless revealed that it scored lower than the hearing group, *t*(224) = 3.192, *p* = .002.

**Table 1:** Participant details: hearing status, deaf group membership, sample sizes, gender and age

Deaf				Hearing
*n* = 106 (boys = 59)				*n* = 120 (boys = 66)
Mean age = 8;10				Mean age = 8;11
SD = 1;8				SD = 1;6
BSL		Spoken English	SSE	
*n* = 29 (boys = 18)		*n* = 69 (boys = 37)	*n* = 8 (boys = 4)	
Mean age = 9;1		Mean age = 8;6	Mean age = 9;5	
SD: 1;7		SD: 1;7	SD: 1;6	
Native BSL	Non‐native BSL			
*n* = 9 (boys = 6)	*n* = 20 (boys = 12)			
Age = 8;1	Age = 9;6			
SD = 0;9	SD = 1;7			

Note: BSL, British Sign Language; SSE, Sign‐Supported English.

The majority of deaf children were severely (*n* = 31) or profoundly (*n* = 54) deaf. Two were mildly and 14 moderately deaf, with data missing from five children. Seventy children used a hearing aid, and 39 a CI (this adds up to more than the 106 children in the group because some children had both). For those children with a CI, the mean age of implantation was 3;3 and ranged between 3 months and 10 years of age (SD = 1;10).

A subgroup of 70 deaf and 74 hearing participants were tested a second time, an average of 21 months (SD = 2 months) after first testing. The mean age of the deaf group at retest was 10;2 (SD = 1;8) and of the hearing group was 10;5 (SD = 1;6).

### Procedure

The study was approved by the UCL Research Ethics Committee. Informed consent was obtained from all participating families prior to testing, and children gave verbal consent with the option to opt out at any time during the testing session.

Testing took place in a quiet room in either the child's school or home. Each session was video recorded and lasted between 60 and 75 min. Children could opt to take short breaks when necessary. Children were assessed by one of two lead researchers, who were supported by a research assistant. One lead researcher was a hearing native user of BSL and their research assistant was a deaf native signer, both very experienced in communicating with deaf children. These researchers used BSL to present all task instructions to deaf children for whom BSL was the preferred language. The second lead researcher and research assistant, both hearing but with competent signing skills, tested all hearing children and deaf children whose preferred language was spoken English or SSE.

### Tasks

#### Semantic Fluency task

The category ‘animals’ was used for the Semantic Fluency task. The instructions were straightforward: ‘Please tell me the names of as many animals as you can. Be as quick as possible. You have one minute. Ready? Go.’ It was rarely necessary to give examples, but when a child seemed unsure a couple of examples (cat and dog) were given. These items were then excluded if the child repeated them during the task. Instructions were given in spoken English, BSL or SSE, depending on the language choice of the child.

#### Expressive One‐Word Picture Vocabulary Test (EOWPVT)

Single word production was tested using the EOWPVT (Brownell 2000) following the standardized administration guidelines. Children are required to name single pictures (mostly simple nouns, e.g., ‘goat’, but also verbs, e.g., ‘writing’, and category labels, e.g., ‘lights’). The test was adapted by substituting three of the test items with alternative pictures to make it more suitable for children in the UK (e.g., ‘badger’ replaced ‘raccoon’). Kyle *et al*. (2016) previously ascertained appropriate signed responses (in BSL); however, in order to ensure that the EOWPVT could be used to assess the vocabulary of both hearing and signing deaf children, 15 test items that do not exist in BSL (e.g., ‘cactus’, ‘banjo’) were removed after administration and an adjusted EOWPVT score was calculated for analysis that excluded these items.

Six EF tasks were chosen for their low language demands, meaning they were less likely to disadvantage children with low language levels.

#### Odd One Out Span

The Odd One Out Span (Henry 2001) is a measure of executive‐loaded visuospatial working memory. The child is instructed to identify which shape is the odd one out and remember its location. At the end of a trial, the child has to recall the location of all of the odd shapes by pointing to the correct box in a sequence of empty grids. There are four trials within a block, beginning with one item to recall. Each block of trials increases in the number of shape locations to recall, with a maximum of six. The test is terminated when two errors are made within the same block. A score is calculated by totalling the number of correctly recalled shape locations.

#### Backwards Spatial Span task

The Backwards Spatial Span task (Wechsler Nonverbal Scale of Ability; Wechsler and Naglieri 2006) is also a test of executive‐loaded visuospatial working memory. The experimenter taps a sequence of blocks and the child is instructed to tap this sequence in reverse. Each trial increases the number of blocks in the sequence to a maximum of nine. The test is terminated after two errors at the same span length, and scored by tallying the number of correct sequences.

#### Design Fluency task

The Design Fluency task (NEPSY; Korkman *et al*. 1998) contains a series of dot arrays. Children are required to generate as many different designs as possible in 1 min by connecting two or more dots with straight lines. The assessment measures visuospatial cognitive fluency and is scored by adding the total number of original designs.

#### Children's Colour Trails Test 1 and 2

The Children's Colour Trails Test 1 and 2 (Llorente *et al*. 2003) is a test of cognitive shifting. For test 1, the children are timed drawing a line connecting the numbered circles from 1 to 15. In test 2, two sets of numbered circles are printed, one set filled with pink and the other yellow. Children are required to join the numbers in ascending order, alternating between colours. In this study, an interference score was calculated, showing the additional time taken in test 2.

#### Tower of London

The Tower of London is a simplified version of the Tower of Hanoi task (Shallice 1982) that measures executive planning. The child needs to move coloured disks from their initial formation, one by one, to match a target configuration. The task was presented using Psychology Experiment Building Language (PEBL) version 0.14 (Mueller and Piper 2014) via a laptop. The first trial was used as an example, and the children continued to complete the seven trials that followed. To score the task, the number of additional moves was calculated by subtracting the minimum number of possible moves from the total number made.

#### Simon Task

The Simon Task (Simon 1990) is a measure of cognitive inhibitory control. On each trial either a sun or an apple appears on the computer screen either left or right of centre. The children are instructed to respond by pressing a key with an apple sticker on the left‐hand side of the keyboard when they see an apple appear, or a pressing a key with a sun sticker on the right‐hand side when they see a sun appear. Each stimulus appears for 750 ms, and the order of trials was randomized for each child. There were 16 congruent (picture on the same side as the response) and 16 incongruent (picture on the opposite side of the response) trials. An interference score was calculated by subtracting congruent from incongruent scores.

### Coding of semantic fluency responses

Spoken responses were transcribed into written English and BSL signs were glossed into written English lexical equivalents. Responses were timed (i.e., it was noted how many seconds into the minute they were produced) so that they could be allocated to quadrants of the minute (i.e., 0–15, 15–30, 30–45 and 45–60 s), and they were coded as correct/incorrect by the first, second and third authors working together. Each incorrect response was coded as one of three types, and these categories fully captured all the errors:

- Repetition of an item.
- Intrusion (i.e., an item that did not fit well into the category ‘animals’, e.g., ‘you’, ‘Loch Ness monster’, ‘calamari’, ‘robot’).
- Unintelligible.

Correct and repeated responses were coded according to semantic clustering. A cluster was defined as two or more adjacent responses that were semantically closely related in some way. We allowed categories to emerge from the data, rather than imposing them. Animal categories included (but were not limited to) ‘zoo’, ‘pet’, ‘farm’, ‘water’, ‘invertebrate’, ‘bird’ and ‘British wild’.

Certain responses potentially fell into more than one category. For example, ‘duck’ could fall into the categories ‘farm’, ‘bird’ or ‘water’, depending on which items it occurred with. ‘Duck’ was coded as ‘farm animal’ when it occurred in the sequence ‘horse–duck–pig–goose’, ‘bird’ when it occurred in the sequence ‘duck–swan–blackbird–robin’ and ‘water animal’ when it occurred in the sequence ‘duck–frog–tadpole’. In assigning categories we endeavoured to be as inclusive as possible, meaning that we tried to ensure that as many responses as possible fell within clusters.

The third author coded all the clusters. The first author then independently coded approximately 10% of the data (from 11 deaf children and 12 hearing children). Interrater agreement of each items for cluster membership was 88.60% of the deaf children's data and 89.04% for the hearing children's data, which is very close to the 88.71% interrater agreement reported by Marshall *et al*. (2013).

## Results

This section is divided into three parts. The first considers the semantic fluency data from time 1 in detail, with respect to the heterogeneity of deaf participants’ language experience and characteristics of fluency output (including error types, clustering, switches between clusters, tapering of responses over time, and the most frequent responses). In the second, the relationship between semantic fluency and the Expressive One‐Word Vocabulary and EF tests is investigated. In the third, the number of correct responses at time 2 and the changes in group means from time 1 to time 2 are presented.

### Semantic fluency data at time 1

**Figure 1:**
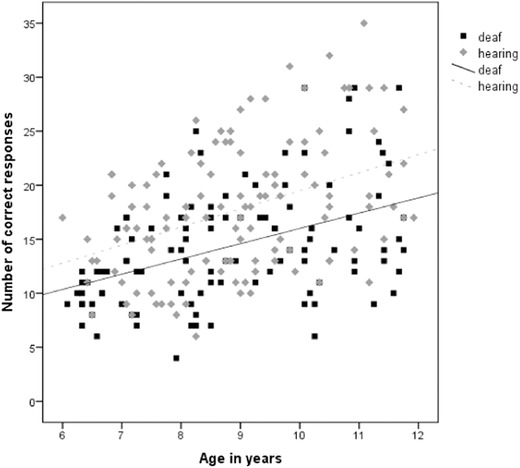
Scatterplot showing the association between the correct number of responses and age for the deaf and the hearing groups.

The number of correct responses was moderately correlated with age for both the deaf and the hearing groups, *r*(106) = .439, *p* < .001 and *r*(120) = .411, *p* < .001 respectively, as shown in figure 1.

Table 2 presents the results of the semantic fluency analysis for the deaf and hearing groups. Independent samples *t*‐tests revealed that despite some overlap in the range of ability, the hearing group significantly outperformed the deaf group with respect to the mean total number of responses, mean number of correct responses, mean number of responses in each quadrant of the minute, mean number of switches, and mean number of clusters. There were no group differences for any of the error types (there were very few errors in either group, with a mean of less than one error per participant) or for cluster size.

**Table 2:** Semantic fluency results for the deaf and hearing groups

		Group					
		Deaf		Hearing			
Variables		mean	SD	Mean	SD	*t*	*p*
Total number of responses		15.15	5.64	18.24	6.28	3.873	< 0.001
Number of correct responses		14.33	5.45	17.63	6.05	4.279	< 0.001
Error types	Repetitions	0.54	0.90	0.38	0.72	1.431	0.154
	Intrusions	0.15	0.66	0.15	0.51	0.012	0.990
	Unintelligible	0.13	0.37	0.09	0.37	0.826	0.409
Correct responses per quadrant	0–15 s	6.38	2.47	7.56	2.48	3.678	< 0.001
	15–30 s	3.76	1.82	4.28	2.35	1.809	0.020
	30–45 s	2.75	1.70	3.30	1.71	2.441	0.031
	45–60 s	2.24	1.70	3.06	2.10	3.202	< 0.001
Clusters	Number of switches	5.41	3.08	6.33	2.75	2.392	0.018
	Number of clusters	3.89	1.77	4.86	1.88	3.985	< 0.001
	Average size of clusters	3.63	1.85	3.38	1.03	1.306	0.193

In order to understand whether fluency performance in each the two groups was related to the production of a greater number of clusters or to the production of bigger clusters, we ran correlations between the number of correct items and the number of clusters, number of switches, and cluster size for the deaf and hearing groups separately. For the deaf group, productivity was strongly related to the number of clusters, *r*(106) = .780, *p* < .001, and to the number of switches, *r*(106) = .648, *p* < .001, but not to cluster size, *r*(106) = –.056, *p* = .568. The same pattern was found for the hearing group: productivity was strongly related to the number of clusters, *r*(120) = .794, *p* < .001, and to the number of switches, *r*(120) = .665, *p* < .001, but not to cluster size, *r*(120) = .110, *p* = .231. Thus it is the production of more clusters, not bigger clusters, that drives productivity in both groups.

Next, the performance of the subgroups of deaf children was analysed. Table 3 presents the semantic fluency data for the deaf group divided into those who responded using BSL, those who used spoken English, and those who used SSE. Because these smaller subgroups were not as well matched for age to the hearing group as the entire deaf group had been (table 1), we partialled out age in an analysis of covariance (ANCOVA). Table 3 therefore reports estimated marginal means and estimated standard error. Pairwise comparisons (Bonferroni corrected) were also computed comparing each of the deaf groups with one another and with the hearing group. These comparisons revealed no significant differences between any of the deaf groups on any of the variables (all *p*s > .05), and for the sake of keeping table 6 as simple as possible, those null results are not reported. Therefore, while hearing status predicts performance on the fluency task (table 6), the type of language used by the deaf children does not.

**Table 3:** Semantic fluency results for the three deaf (BSL, spoken English and SSE) groups and the hearing group

		Group									
		Deaf									
		BSL		Spoken English		SSE		Hearing			
Variables		e.m. mean	e. SE	e.m. mean	e. SE	e.m. mean	e. SE	e.m. mean	e. SE	Pairwise comparisons with hearing group	*p*
Total number of responses		14.35	1.00	15.85	0.65	12.84	1.91	18.19	0.49	BSL**	0.004
										Spoken English*	0.028
										SSE*	0.044
Number of correct responses		13.45	0.97	15.10	0.63	11.65	1.85	17.57	0.47	BSL**	0.001
										Spoken English *	0.012
										SSE*	0.013
Different error types	Repetitions	0.58	0.15	0.55	0.10	0.35	0.29	0.38	0.07	BSL	1.000
										Spoken English	1.000
										SSE	1.000
	Intrusions	0.12	0.11	0.09	0.07	0.84	0.20	0.15	0.05	BSL	1.000
										Spoken English	1.000
										SSE*	0.006
	Unintelligible	0.21	0.07	0.12	0.04	0.002	0.13	0.09	0.03	BSL	0.769
										Spoken English	1.000
										SSE	1.000
Correct responses per quadrant	0–15 s	5.58	0.41	6.67	0.27	5.12	0.78	7.39	0.20	BSL**	0.001
										Spoken English	0.198
										SSE*	0.031
	15–30 s	3.58	0.38	3.53	0.25	3.02	0.73	4.15	0.19	BSL	1.000
										Spoken English	0.296
										SSE	0.815
	30–45 s	2.51	0.31	2.62	0.20	2.71	0.59	3.08	0.15	BSL	0.625
										Spoken English	0.439
										SSE	1.000
	45–60 s	1.74	0.34	2.30	0.22	0.68	0.64	2.95	0.17	BSL**	0.008
										Spoken English	0.106
										SSE**	0.004
Clusters	Number of switches	5.69	0.51	5.37	0.33	4.99	0.98	6.31	0.25	BSL	1.000
										Spoken English	0.148
										SSE	1.000
	Number of clusters	3.95	0.32	3.94	0.21	3.37	0.61	4.85	0.16	BSL	0.078
										Spoken English **	0.004
										SSE	0.121
	Average size of clusters	3.20	0.27	3.87	0.18	3.10	0.52	3.38	0.13	BSL	1.000
										Spoken English	0.160
										SSE	1.000

Notes: BSL, British Sign Language; SSE, Sign‐Supported English; e.m. mean, estimated marginal mean; e. SE, estimated standard error.

^*^ *p* < .05, ^**^ *p* < .01, ^***^ *p* < .001.

In table 4 we report the data for the native and non‐native signers. Again, because the groups were not well matched for age, we partialled out age in an ANCOVA and report estimated marginal means and estimated standard error. The data must be treated with caution because of the small number of native signers (*n* = 9), but findings indicate that the native signers produced more items overall and more correct items. No other comparisons reached statistical significance.

**Table 4:** Semantic fluency results for the deaf native and non‐native users of BSL

		Deaf BSL					
		Native		Non‐native			
Variables		e.m. mean	e. SE	e.m. mean	e. SE	*F*	*p*
Total number of responses		16.93	1.15	13.88	0.74	4.545*	0.043
Number of correct responses		15.93	1.11	12.98	0.72	4.573*	0.042
Different error types	Repetitions	0.72	0.31	0.53	0.20	0.256	0.617
	Intrusions	0.19	0.16	0.12	0.10	0.124	0.728
	Unintelligible	0.10	0.15	0.25	0.10	0.637	0.432
Correct responses per quadrant	0–15 s	6.41	0.62	5.47	0.40	1.501	0.231
	15–30 s	3.91	0.63	3.59	0.41	0.160	0.692
	30–45 s	3.32	0.45	2.26	0.29	3.595	0.069
	45–60 s	2.22	0.51	1.65	0.33	0.808	0.377
Clusters	Number of switches	5.80	0.90	5.89	0.58	0.007	0.932
	Number of clusters	4.96	0.53	3.67	0.34	3.825	0.061
	Average size of clusters	3.75	0.48	2.94	0.31	1.928	0.117

Note: ^*^ *p* < .05, ^**^ *p* < .01, ^***^ *p* < .001.

**Figure 2:**
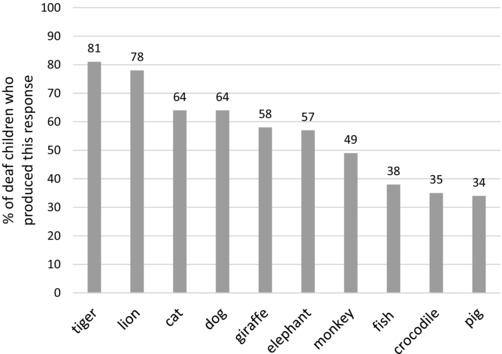
Most frequent responses from the deaf group (all responses given by 33% or more of the group).

**Figure 3:**
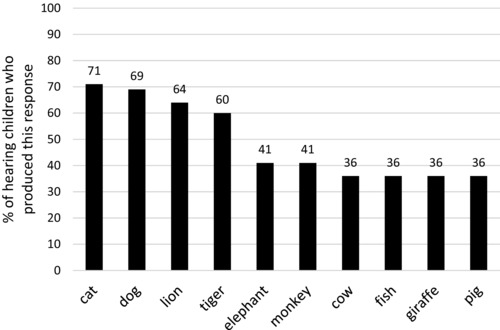
Most frequent responses from the hearing group (all responses given by 33% or more of the group).

Next we consider the nature of the lexical items produced by the deaf group as a whole and by the hearing group. The deaf children produced 196 different types of animals, and the hearing children produced 297. Figures 2 and 3 show the responses which were produced by 33% or more of the children in each group (following Marshall *et al*. 2013). For each group there are 10 such responses, and of those, nine were produced by both groups (‘cat’, ‘dog’, ‘elephant’, ‘fish’, ‘giraffe’, ‘lion’, ‘monkey’, ‘pig’, ‘tiger’). A positive association between lexical frequency and the frequency of responses in the fluency task would be predicted, but is rarely investigated. In order to determine whether a lexical frequency effect exists in deaf children's responses and is similar to magnitude to any effect found in hearing children, the frequency of the full set of responses in the two groups was correlated with the log of their lexical frequencies as reported in the CELEX database (Baayen *et al*. 1995). For both groups, a moderate effect of lexical frequency was found that was very similar in magnitude for the deaf children, *r_s_*(155) = .522, *p* < .001, and for the hearing children, *r_s_*(208) = .554, *p* < .001.

Finally in this part of the results section, table 5 presents the percentile scores for the deaf children's number of correct responses, broken down by 2‐year age bands. The aim of table 5 is to provide normative data should clinicians or researchers wish to use the semantic fluency test with deaf children in the 6–11 age group. As there were no significant differences in performance among the deaf subgroups, normative data for the whole deaf group are reported.

**Table 5:** Age band percentile scores^a^ for deaf participants’ semantic fluency

				Percentile scores												
Age band (years)	*N*	Mean (SD)	Minimum–maximum	1st	2nd	5th	10th	20th	30th	40th	50th	60th	70th	80th	90th	95th
6–7	37	11.65 (4.16)	4–23	4	4	6	7	8	9	10	11	12	13	15	17	21
8–9	39	14.87 (4.51)	7–25	7	7	7	8	11	12	13	16	17	17	18	21	23
10–11	30	16.93 (6.54)	6–29	6	6	8	9	12	13	14	15	17	20	24	28	29

Note: ^a^Scores are rounded to the nearest whole number.

### Relationships between semantic fluency, expressive vocabulary and executive function

In this second part of the results section, the relationships between semantic fluency and the EOWPVT and EF tasks are investigated. The group comparisons between the deaf and hearing groups for the EOWPVT and EF tasks were reported in Botting *et al*. (2016). To summarize the results of that paper, the hearing group significantly outperformed the deaf group on all measures except for design fluency.^1^

Table 6 presents the partial correlations (controlling for age) between the number of correct items produced in the semantic fluency task, and the scores for the individual EF tasks and the EOWPVT. Given the group differences in *T*‐scores on the Wechsler Abbreviated Scale of Intelligence (WASI) matrix reasoning task identified in the Participants section, partial correlations between WASI scores and semantic fluency are also presented. Correlations are reported for the deaf and hearing groups separately, and for all the children combined. EOWPVT, the two working memory tasks (Odd One Out and Backwards Spans) and the Design Fluency task correlated most strongly with semantic fluency in both groups separately and the two groups combined. Tower of London performance correlated significantly with semantic fluency in the deaf group but not for the hearing group. WASI matrix reasoning score correlated significantly with semantic fluency in both groups and the two groups combined.

**Table 6:** Partial correlations (controlling for age) between semantic fluency and each EF task or vocabulary task

	Deaf		Hearing		All children	
	*r*	*p*	*r*	*p*	*r*	*p*
Working memory: Odd One Out Span	.443***	< .001	.450***	< .001	.500***	< .001
Working memory: Backwards Spatial Span	.409***	< .001	.254*	.013	.400***	< .001
Non‐verbal fluency: Design Fluency task	.383***	< .001	.421***	< .001	.474***	< .001
Cognitive flexibility: Colour Trails Test	−.169	.103	−.002	.986	–.167*	.021
Planning: Tower of London	–.404***	< .001	–.174	.092	–.327***	< .001
Inhibition: Simon Task	.097	.353	.048	.645	.126	.083
Expressive vocabulary: EOWPVT	.565***	< .001	.493***	< .001	.592***	< .001
WASI: matrix reasoning	.321**	.001	.360***	< .001	.376***	< .001

Notes: EOWPVT, Expressive One‐Word Picture Vocabulary Test (EOWPVT); WASI, Wechsler Abbreviated Scale of Intelligence.

^*^ *p* < .05, ^**^ *p* < .01, ^***^ *p* < .001.

In order to investigate further the relationship between these variables, *z*‐scores for the EF tasks (which correlated sufficiently highly with one another) were calculated and combined into a single, composite, score, as was done in the study by Botting *et al*. (2016). Regression analyses were then carried out with semantic fluency scores as the dependent variable, and age, matrix reasoning, vocabulary score, the EF composite score, and group (deaf or hearing) as the predictors. Age and matrix reasoning scores were entered simultaneously in the first block, then vocabulary and EF composite scores simultaneously in the second block, and finally group in the third block.

The model with just age and matrix reasoning was significant, *F*_(2,188)_ = 33.053, *p* < .001. This model accounted for 26.2% of the variance in semantic fluency scores. Both variables were significant predictors; age: *Beta* = .426, *t* = 6.685, *p* < .001; matrix reasoning: *Beta* = .359, *t* = 5.635, *p* < .001. Adding vocabulary and EF composite scores to the model explained an additional 23.4% of the variance, *F*_(4,188)_ = 45.354, *p* < .001. Both vocabulary and EF composite scores were significant predictors in this model; vocabulary: *Beta* = .381, *t* = 5.272, *p* < .001; EF composite: *Beta* = .314, *t* = 3.982, *p* < .001. The third model with group added, however, did not explain any additional variance (0.0%) in semantic fluency scores.

Repeating the same regression analysis on the deaf and hearing group separately revealed exactly the same pattern. The results demonstrate that, alongside age and non‐verbal reasoning skills, EF and vocabulary scores were both unique and significant predictors of semantic fluency scores in both groups.

### Semantic fluency data at time 2

**Figure 4:**
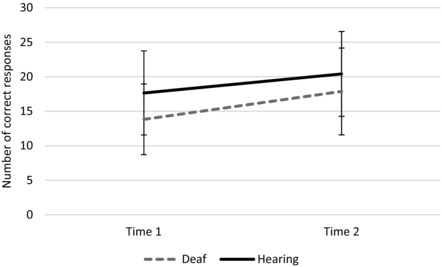
Correct responses on the semantic fluency task at times 1 and 2 (vertical bars indicate standard deviations).

The majority of the participants (70 deaf and 74 hearing) were retested on the semantic fluency task nearly 2 years later. For this analysis, the data for the deaf children were not subgrouped by language use (BSL, spoken English or SSE) because of its lack of effect on semantic fluency at time 1. Figure 4 presents the mean number of correct responses for each group at time 1 and time 2. A 2 × 2 ANOVA, with time (Time 1, Time 2) as the within‐subjects factor and group (Deaf, Hearing) as the between‐subjects factor revealed a significant effect of time, *F*_(1,142)_ = 68.208, *p* < .001, partial eta squared = .324 (a large effect size; Cohen 1988), and of group, *F*_(1,142)_ = 12.470, *p* = .001, partial eta squared = .081 (a medium effect size). These analyses indicate that children produced significantly more correct responses at time 2 compared with time 1, and that the hearing children produced significantly more correct responses than the deaf children. The interaction between time and group was not significant, *F*_(1,142)_ = 2.440, *p* = .120, partial eta squared = .017 (a small effect size), indicating that the gap between the two groups did not change over time.

## Discussion

The aims of this study were to investigate semantic fluency in deaf children aged 6–11 by comparing deaf and hearing children's lexical retrieval patterns, and by comparing the responses of deaf children who used BSL with those who used spoken English and SSE. We investigated how semantic fluency performance is related to children's expressive vocabulary and EF skills, and we also tested the semantic fluency of a subset of the participants nearly 2 years later, in order to investigate how much progress they had made in that period.

The semantic fluency category used in this study, as in many others, was ‘animals’. Deaf children produced fewer responses than hearing children of the same age, and this was the case for all four quadrants of the minute. A further difference was that deaf children drew on a smaller set of lexical items than hearing children. However, there were also similarities: neither group produced many errors (repetitions, intrusions, and unintelligible responses), average cluster size did not differ significantly between the two groups, both groups shared nine of their ten most frequent responses (cat, dog, elephant, fish, giraffe, lion, monkey, pig, tiger), and both groups showed a significant correlation between response frequency and the log of lexical frequencies reported in the CELEX database (Baayen *et al*. 1995). For both groups, productivity was driven by cluster number and the number of switches rather than cluster size.

Our deaf group was heterogeneous with respect to language experience, and we sought to understand the effect of language mode on semantic fluency performance by comparing the performance of children who responded using BSL, spoken English and SSE. The sample size of the group who used SSE was small, so their results should be treated with caution. Nevertheless, whether children used BSL, spoken English or SSE seemed to have no influence on their semantic fluency performance: all produced fewer responses than the hearing children, but did not differ from one another. Within the signing group, however, native signers (i.e., children who had been exposed to BSL from birth) produced more items than non‐native signers (i.e., children who had only been exposed to BSL later in childhood). Hence although the type of language used does not appear to influence fluency performance, language proficiency does. Again, these results must be treated with caution because of the small sample size of the native signer group. Nevertheless, that language proficiency affects fluency performance is consistent with the results of our finding that expressive vocabulary in either spoken English or BSL is a significant predictor of semantic fluency scores. Our data suggest that deaf children generate fewer items than hearing children partly because they have a smaller pool of items to draw from in their lexicon. Furthermore, we have also shown that semantic fluency performance is related to a composite of EF tasks that included the Design Fluency task, Working Memory task and the Tower of London. Previous work on hearing populations has shown that semantic fluency requires both vocabulary and EFs (e.g., Ardila *et al*. 2006, Bose *et al*. 2017), and our data directly support the same finding for deaf children, indicating that semantic fluency is measuring equivalent cognitive abilities and has construct validity across both groups.

Our final analysis compared semantic fluency performance in a subset of children at two different testing times, 21 months apart. Both groups produced more responses at time 2 compared with time 1, showing development over the course of the study. There was no interaction between group and time, indicating that while the deaf children did not catch up with the hearing children during that time, neither did the gap between them widen. Both groups showed a similar rate of development on the task but the deaf group had a lower starting point.

Our results are consistent with the few studies that have previously investigated semantic fluency in deaf children. As in the study by Wechsler‐Kashi *et al*. (2014) of deaf children with CIs, deaf children in our study produced fewer items compared with hearing children of the same age. With respect to deaf children who used sign, our results replicate the findings of Marshall *et al*. (2013) and Beal‐Alvarez and Figueroa (2017) that the same ‘cognitive signatures’ that characterize children's semantic fluency responses in spoken languages—namely clustering of responses, the slowdown in response rate during the course of the minute, and the production of prototypical items—also characterize responses in a signed language. More cross‐linguistic work on other signed languages is needed, but studies of deaf adults who use ASL (Beal‐Alvarez and Figueroa 2017), Portuguese Sign Language (Moita and Nunes 2017) and Greek Sign Language (Vletsi *et al*. 2012) reveal similar patterns of responses to those found with deaf adults who use BSL (Marshall *et al*. 2014), indicating that, just as the semantic fluency task has utility across different spoken languages (Ardila *et al*. 2006), so it does across signed languages.

Our study provides comprehensive data on deaf children's performance on one specific semantic task—animal fluency—from the largest sample to date, and is the first to consider development on this task over time using a longitudinal paradigm. Limitations are the small numbers of children who were native users of BSL and who used SSE, and the use of just one semantic category (albeit, the most widely used category in semantic fluency research, ‘animals’). Future research is needed to confirm the patterns of responses and to provide normative data for other semantic categories. The results should be treated with appropriate caution because the language‐learning opportunities open to deaf children in the UK are changing rapidly: access to universal newborn hearing screening and advances in CI technology are resulting in improved access to spoken language, but the increase in deaf children being educated in mainstream schools with no specialist provision and no exposure to skilled signers means that they have reduced knowledge of sign language (Consortium for Research in Deaf Education (CRIDE) 2016). This means that the population of deaf children who participated in our study might not be representative of the deaf children in UK primary schools in the future.

## Conclusions

Our findings confirm that semantic fluency is structured in a similar way across spoken and sign languages, and that hearing and deaf children approach the task using the same strategies. This means that a tool that has long been used with the hearing population can be used experimentally to investigate lexical organization in deaf children, and clinically using our normative data to investigate impairments in their language or EFs. A further strength of this study is that it shows that semantic fluency has equivalent validity across groups of deaf children using different forms of spoken and signed communication, thus enabling simpler and more confident assessment of semantic fluency in this highly heterogeneous population.

## Note

1 As explained previously, the sample does not overlap exactly with the sample included in Botting *et al*. (2016), but the same pattern of EF and vocabulary results that they report holds for this sample.
